# WHACS: An Improved Global Wave Hindcast for the Australian Climate Service

**DOI:** 10.1038/s41597-026-06864-6

**Published:** 2026-02-21

**Authors:** Grant Smith, Alberto Meucci, Claire Spillman, Ron Hoeke, Vanessa Hernaman, Claire Trenham, Stefan Zieger, Bryan Hally, Emilio Echevarria

**Affiliations:** 1 Bureau of Meteorology, Melbourne, VIC Australia; 2 CSIRO Environment, Aspendale, VIC Australia; 3 Department of Infrastructure Engineering, The University of Melbourne, Melbourne, VIC Australia

**Keywords:** Physical oceanography, Physical oceanography

## Abstract

A multi-decadal global wind-wave hindcast dataset—WHACS: the Wave Hindcast for ACS—spanning 1979 to near present was developed to offer insight into historical wave conditions both directly and as boundary forcing to localised simulations. Applications for WHACS include coastal management, climate research, and renewable energy projects, ultimately helping communities and industries make informed decisions to improve safety, efficiency, and resilience regarding wave conditions. This dataset features a near-global spherical multi-cell (SMC) grid that aligns with the Bureau operational wave forecast model and has been calibrated to better represent extreme wave conditions by improving the representation of extreme winds. Spanning from 1979 to near present, WHACS available output consists of multiple hourly bulk and spectral partition wave parameters for the native SMC grid, as well as regular global and regional regridded bulk wave parameters. For the Indo-Pacific, a gridded output of full spectral data is available across exclusive economic zones.

## Background & Summary

Wave hindcast data products provide consistent estimates of historical ocean surface gravity wave conditions with high temporal and spatial coverage. Wave hindcasts are analogous to reanalysis products, but without the use of any wave observations and data assimilation. As part of the Australian Climate Service (ACS), a new global wave hindcast data product was developed known as WHACS (Wave Hindcast for ACS)^1^. It spans 1979 to near present and is updated monthly. It is a replacement for the existing Collaboration for Australian Weather and Climate Research (CAWCR) Wave Hindcast^2^, first developed in 2010^3^. This legacy wave hindcast has proved highly valuable in providing full wave spectral information for coastal hazard assessments^4,5^, offshore and coastal engineering design^6^, renewable energy projects^7–9^, climatology studies^10,11^, and other wave model downscaling work that requires boundary conditions from a global or basin scale model^12,13^.

The ACS was established in 2021 to increase Australia’s capability to better prepare for, respond to, recover from, and adapt to a more challenging climate and natural hazards. To achieve this, the focus is on improving access to integrated trusted data, information and expert advice, together with building and enhancing Australia’s climate and natural hazard intelligence capability (www.acs.gov.au). ACS partners are the Bureau of Meteorology, CSIRO, Geoscience Australia, and the Australian Bureau of Statistics. A primary task of the ACS is to deliver climate and natural disaster risk information to the National Emergency Management Agency (NEMA), Department of Climate Change, Energy, the Environment and Water (DCCEEW), and the National Climate Risk Assessment (NCRA). Coastal hazards have been recognised as critical for Australia’s adaptation and resilience to future climate change and are identified as a priority area within ACS. Coastal hazards driven by extreme wave activity in conjunction with extreme sea levels can lead to erosion and inundation, posing significant risks to communities by creating accessibility issues and damaging critical infrastructure. The frequency, intensity and magnitude of coastal hazards are expected to increase in the coming decades^14^.

Wave climate studies require long-term time series datasets spanning multiple decades to capture interdecadal variability, and these datasets need to be regularly updated for ongoing assessments. Relying only on observational records can be limiting across geospatial extents, and instrument failures can introduce gaps and spurious signals over time. A gridded hindcast dataset such as WHACS offers consistent spatiotemporal coverage, providing a comprehensive picture of wave climate across the decades. WHACS delivers this through improved model physics and enhanced coastal resolution.

In addition, WHACS was also developed to provide spectral boundary conditions for the ACS downscaled coupled wave and hydrodynamic model, the Coupled Coastal Hazard Prediction System (CCHaPS). The CCHaPS coastal modelling system generated a historical water level dataset for Australia for the purpose of assessing extreme events and provide an up-to-date baseline for coastal climate projection assessments as part of ACS objectives^15^. CCHaPS simulates extreme coastal water levels resulting from processes such as astronomical tides, storm surge, wave setup and runup, sea level variability, and sea-level rise. To accurately represent these extremes, especially along the Australian coastline where long-period swells from distant storms in the Pacific, Indian, and Southern Oceans are dominant, CCHaPS requires wave frequency–direction spectra as offshore boundary conditions. WHACS plays a critical role by providing these inputs through a near-global wave hindcast that is properly calibrated for extreme events, ensuring reliable simulations of coastal hazards and extreme sea levels.

The new wave hindcast WHACS dataset can also be used in the analysis of past events to support any after-action reviews into government responses and improved integrated disaster management. The legacy CAWCR wave hindcast has seen high utilisation; WHACS provides a performance and technological update. WHACS also has the added benefit of aligning with the Bureau’s operational wave forecast model AUSWAVE-G4^16^. Statistics derived from the hindcast will provide consistent context for operationally forecasted events and extremes.

WHACS also provides spectral data across the globe at 11,260 selected points, ranging from observational network locations and finely gridded distributions across nearshore, shelf, and islands in the Indo-Pacific (and includes the boundary forcing for CCHaPS). Bulk and spectral partition wave parameters are available across both native (“spherical multiple-cell” or SMC) and rectilinear grids for Australia and the world.

## Methods

This dataset consists of structured outputs from a spectral wave model. Spectral wave models predict phase-averaged wind waves from the open ocean to the coast for various wind speeds, accounting for propagation, refraction, dissipation and non-linear wave-wave interactions. Tolman *et al*.^17^ highlighted the importance of biases in global wave hindcasts due to biases in wind speed, particularly in the Southern Ocean, and the absence of swell decay terms. Tolman *et al*.^17^ improved operational wave modelling with the release of WAVEWATCH III® (hereafter WW3) version 4.18 and included the observation-based physics ST6 and spectral sink terms to represent swell decay for ST4^18^ and ST6^19^ physics. The release featured new capabilities for multiple-resolution grids (i.e. SMC and unstructured), computational efficiencies, and improved support for NetCDF file format. Multiple-resolution grids eliminate the need for a nested multi-grid approach for the transition of the wave field from the open ocean to the coast and significantly improve computational efficiency.

The legacy CAWCR wave hindcast was originally developed in 2008 with WW3 v4.08, then upgraded to v4.18b in 2013^2^. The WHACS hindcast uses the latest version release 6.07^20^, which includes some model advancements over v4.18b such as updated physics (e.g. upgrades for ST6 for improved energy levels at higher frequencies, Liu *et al*.^21^), new capabilities such as coupling module interface, implicit numerical scheme and domain decomposition for unstructured grids^20^.

**Fig. 1:**
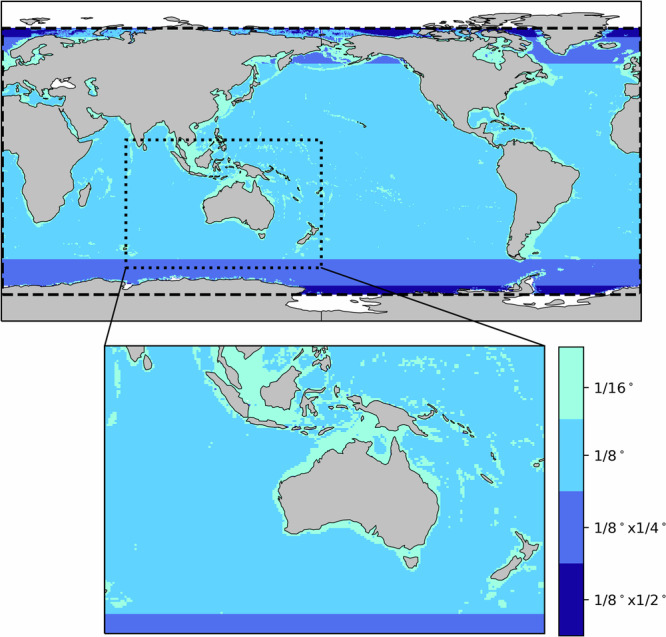
Global view of the Spherical Multiple-Cell (SMC) grid (top panel) and an enhanced view of the grid surrounding Australia (bottom panel), highlighting the varying resolution from 1/8° in open seas to 1/16° in shelf regions. A dashed perimeter spanning most of the globe delineates the domain for the rectilinear regridded output for bulk parameters at 1/8° resolution. Dotted perimeter around the Australasian region shows the finer resolution bulk parameter rectilinear regrid at 1/16°.

The wave model configuration and bathymetry grid were adopted from the Bureau’s operational wave forecast system, AUSWAVE^16^, to ensure consistency between the hindcast and operational forecast products. This alignment facilitates the seamless development of climatologies for contextualising forecasts and improving verification. The SMC grid scales to finer resolutions in shelf regions. The resolution ranges from 1/8° (~10 to ~14 km) in open seas to 1/16° (~5 to ~7 km) near the coast, offering sufficient detail to meet boundary input requirements for high-resolution coupled wave-hydrodynamic modelling around Australia (Fig. 1). Whilst evaluation and development were focussed on the Australian region, the SMC configuration is expected to provide similar quality results globally.

To align with the CCHaPS hindcast, which uses atmospheric data from BARRA2^22^—a national climate reanalysis providing winds at 12 km resolution and forced at its lateral boundaries by the ECMWF reanalysis, ERA5^23^—WHACS was configured to use wind and ice data from ERA5. This ensures consistency between the wave characteristics of storm waves generated outside the national model domain in WHACS and local wind-forced storm waves within the CCHaPS domain (using BARRA2 winds). ERA5 is likely the most accurate source of wind forcing for wave modelling due to its superior spatial resolution and extensive assimilation of observational data compared to other global reanalyses (e.g. Fan *et al*.^24^).

Neutral winds are preferably used for wave modelling as they align directly with the applied wind input parameterisations. Unlike conventional winds, neutral winds are not affected by atmospheric stability, making them a more consistent and physically appropriate forcing variable. They are derived from the surface stress and roughness length under the assumption of neutral stratification, ensuring that the wind direction matches the direction of the applied stress. Because neutral winds adjust for stability effects (being slower than real winds in stable conditions and faster in unstable ones), they provide a stability-independent measure of the momentum actually transferred to the surface. The roughness length used in their calculation reflects surface characteristics, such as sea state, further linking the wind forcing to the processes relevant for wave generation. Whilst results showed that neutral winds were generally comparable to conventional winds, some improvement in the western Pacific is expected^25^.

**Fig. 2:**
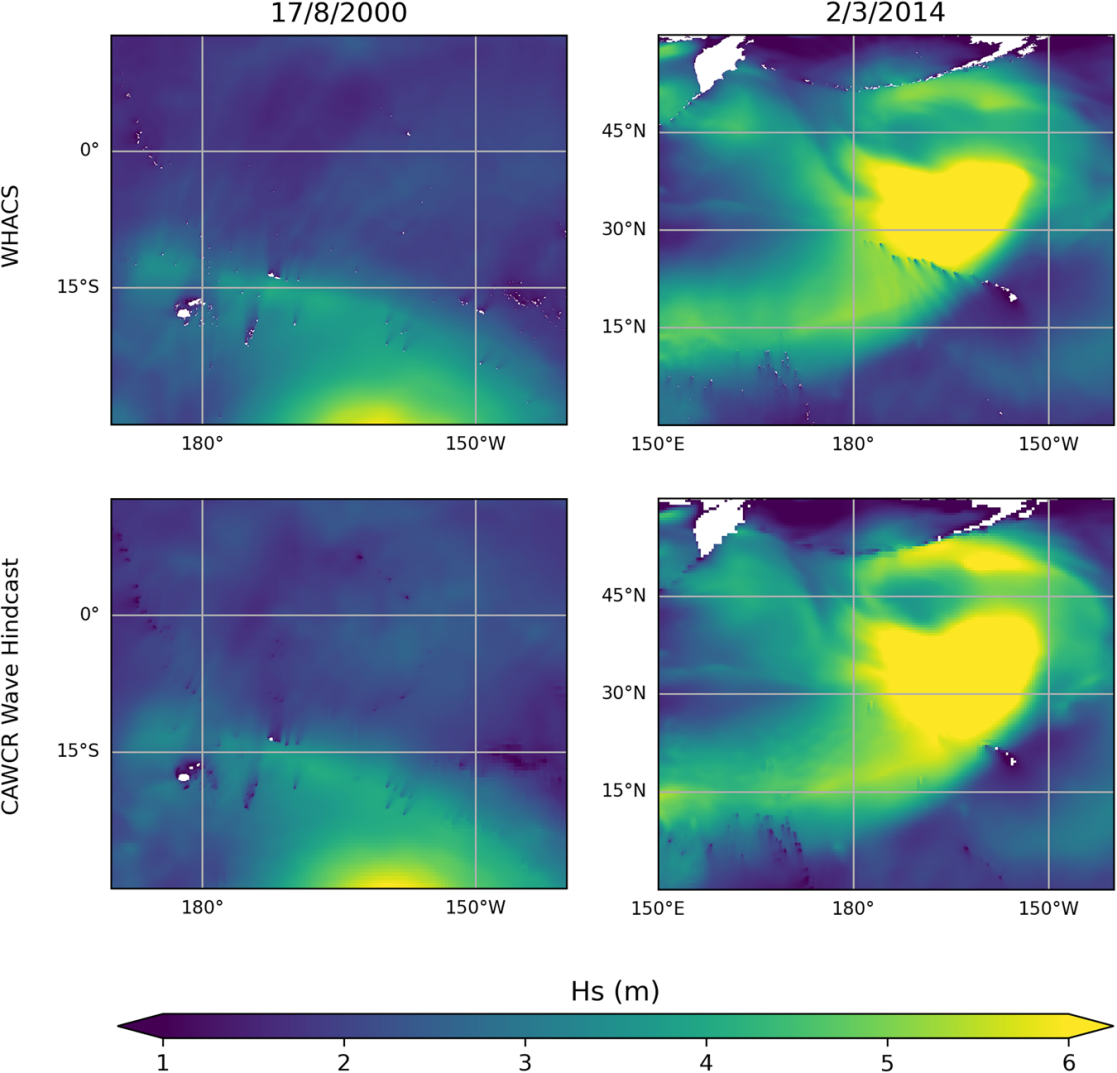
Comparison between the high resolution WHACS grid (top panel) and the high-resolution CAWCR grid (bottom panel) with sub-grid blocking. The snapshot shows the wave field (significant wave height) for a significant wave event in the South Pacific during the “Millennium wave” event for 17th August 2000 (left column), and an event from early March 2014 in the North Pacific (right column).

The CAWCR wave hindcast included subgrid wave blocking to mimic the damping of the wave field from unresolved islands in large-scale wind wave models^26^. WHACS does not include subgrid wave blocking; however, the high-resolution global grid ensures that many small features are well captured. Figure 2 shows the difference between subgrid blocking in the CAWCR hindcast and the WHACS global grid in the north and south Pacific. Enhanced detail can be seen in the blocking and shadowing of the wave field for the Hawaiian Islands Chain (U.S.), the Aleutian Islands, and the Kuril Islands in WHACS. The Gilbert Island group of Kiribati and the Marshall Islands show similar representation between the two hindcasts, with Fiji, Samoa, Tonga, and French Polynesia showing improved detail in WHACS. A small number of minor features are absent in WHACS that can be identified in the CAWCR wave hindcast (represented via subgrid blocking), e.g., some islets and seamounts of Tokelau, Tuvalu, and the Line Islands group of Kiribati.

Inclusion of ocean currents was assessed to determine if any significant hindcast improvements were likely. Daily ocean current information was sourced from the ACCESS-S2 (Australian Community Climate and Earth-System Simulator – Seasonal v2) reanalysis^27^, covering most of the hindcast period (1981 to 2018). Although there exists evidence for improvements in significant wave height in the Southern Ocean by including hourly current data^28^, it was found that improvements in WHACS were minimal, likely due to the daily temporal resolution dampening the sub-daily variability and strength of wind driven currents, especially in the Southern Ocean. As a result, surface current data were not used in the production of WHACS.

Within WW3, the observation-based source term package ST6^19,21,29^ has been recommended as most suitable for wave modelling with an emphasis on extreme events^30,31^. The WW3 physics settings selected for WHACS are provided in Table 1. Spectral discretization was 28 frequencies, with 1.1x increments starting at 0.04118 Hz to 0.524 Hz, and 30 × 12° directional bins. Sea ice dissipation was parameterised with the IC0 approach^26^, with no dissipation for sea ice concentrations <25%, a linear decay between 25% and 75%, and total wave blocking for ice concentrations above 75%.

**Table 1:** WW3 physics settings selected for WHACS. These settings include parameterisations for wind input, wave dissipation, and nonlinear interactions, ensuring consistency with established Australian operational wave modelling frameworks: AUSWAVE-G4^16^.

Identifier	Description
IS0	No scattering by sea ice.
IC0	Simple sea ice blocking.
REF0	No reflection.
LN1	Cavaleri & Malanotte-Rizzoli^51^ with filter for low-frequency energy.
FLX4	Drag coefficient adjustment for ST6^52,53^
ST6	Observation based physics for deep-water source/sink terms^19,29^
MLIM	Use of Miche^54^ style shallow water limiter in equation for maximum wave energy
NL1	Discrete interaction approximation^55^
BT4	Bottom friction according to SHOWEX, see Ardhuin *et al*.^56^
DB1	Battjes & Janssen^57^ depth induced breaking
BS0	No bottom scattering used.
TR0	No triad interactions used.
WNX1	Approximately linear wind speed space interpolation.
WNT1	No time wind interpolation.
WCOR	Wind correction model parameters.

The ERA5 dataset has a known deficiency in that it underestimates strong wind speeds, with average mean differences of −3.7% between 10 to 15 m/s, −6.6% between 15 and 20 m/s, and −9.7% above 20 m/s^32^. The wind correction setting (WCOR; Table 2) in WW3 was utilised to enhance extreme winds to account for a portion of the underestimation using the WCOR1 and WCOR2 parameters:

$${U}_{{neutral}} > {WCOR}1,\,U={U}_{{neutral}}+\left(\left({U}_{{neutral}}-{WCOR}1\right)\ast {WCOR}2\right)$$

**Table 2:** Configuration of ST6 WCOR settings for the correction of ERA5 10-meter surface wind speed. The correction is based on a threshold value (WCOR1) and a correction factor (WCOR2), which vary across different configurations.

ST6 WCOR Configuration	WCOR1	WCOR2
WCORa	23	1.08
WCORb	15	0.15
WCORc	15	0.3
WCORd	20	0.9

There have been recommendations for WCOR parameters in correcting for extreme winds in ERA5 (e.g. Dodet *et al*.^33^; Alday *et al*.^34^; Elshinnawy *et al*.^35^). Elshinnawy *et al*.^35^ provided recommendations for a global wave model using ERA5 with a global resolution of 0.5°. However, the calibration for extreme winds will differ between model resolutions and, therefore, needs to occur on a model-specific basis. A baseline was adopted by Dodet *et al*.^33^ as a starting point for calibration (WCOR1 = 23 m/s, WCOR2 = 1.08).

**Fig. 3:**
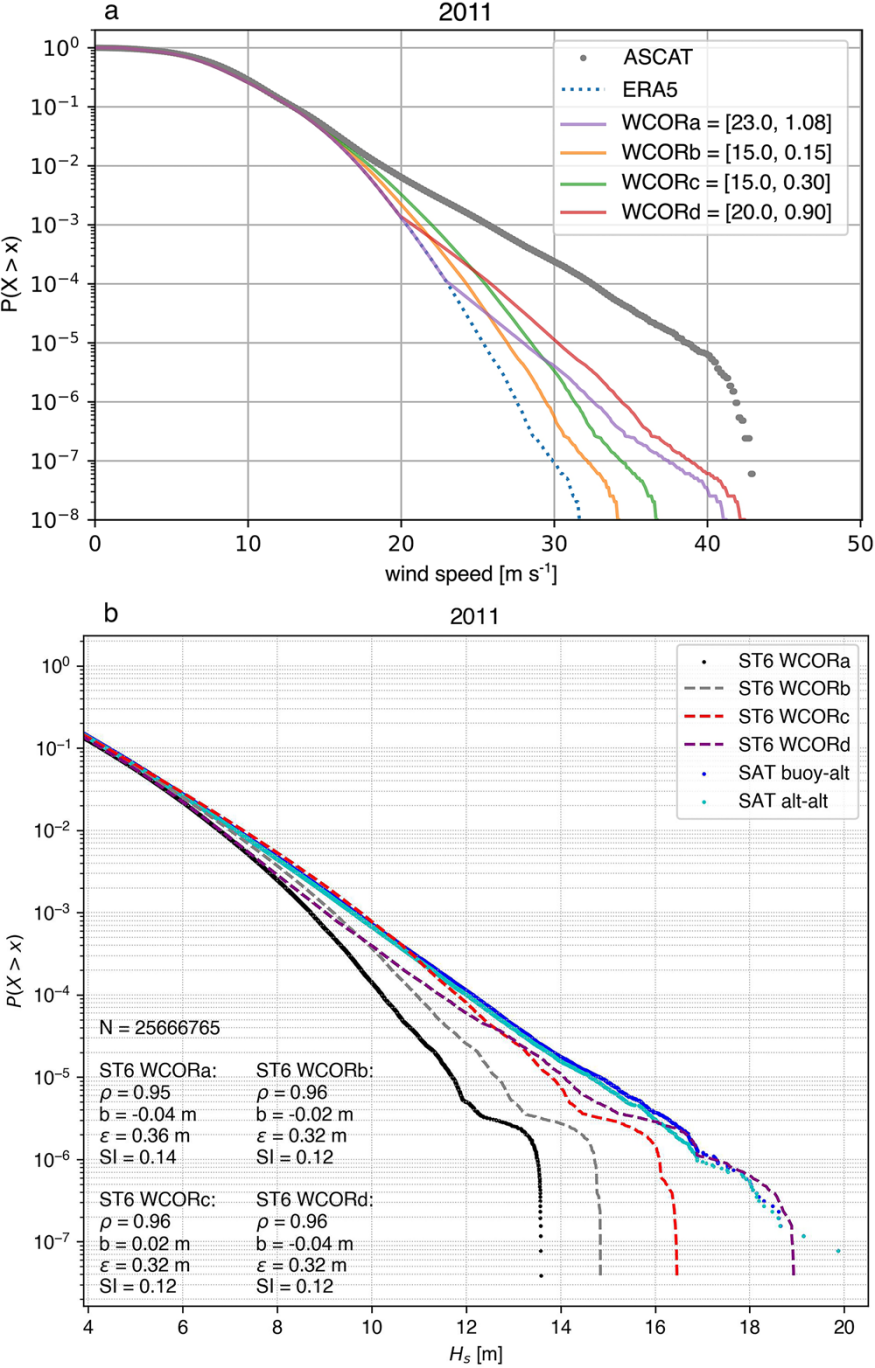
Extreme wind speed and wave heights calibration. (**a**) Probability of Exceedance (PoE) curves for model–scatterometer (ASCAT) collocations of 10-metre surface wind speed for the year 2011. (**b**) PoE curves for significant wave height (Hs) from model–altimeter collocations for 2011, based on the correction tests on the ERA5 wind input. The analysis includes over 25 million collocations at the WHACS model’s 1/8° spatial resolution, using data from the Australian Ocean Data Network (AODN) satellite altimeter archive. Key statistical metrics for evaluation, including correlation (r), bias (**b**), root mean square error (**e**), and scatter index (SI), are displayed in the bottom left corner. Both altimeter-buoy and altimeter-altimeter AODN satellite calibration curves are shown, but the statistics only correspond to the altimeter-altimeter calibration^58^.

The set of correction options that were tested is listed in Table 2. Figure 3 shows how the corrections change the probability of the wind speed events occurring alongside observations taken from the Advanced Scatterometer (ASCAT) satellite product^36^. The wind speed frequencies start to diverge from observations at around 15 m/s (Fig. 3a). However, WHACS calibration tests found that this threshold resulted in a detrimental effect on the bulk of the mid-range wave heights when amplified enough to correct the extremes. It was decided that 20 m/s would be a more appropriate threshold, affecting only the underpredicted larger waves.

A comparison between ERA5 and ASCAT wind measurements using various combinations of correction parameters found that WCOR1 = 20 and WCOR2=0.9 were a suitable candidate that enhanced strong winds without adversely affecting the mean wave error (Fig. 3).

Model runs for 2011 (Fig. 3b) show that uncalibrated ST6 significant wave heights begin to deviate from the observations from 8 m, with a significant drop-off occurring close to 14 m. Optimum calibration (ST6 WCORd) adheres closely to the observations up to H_s_ of 19 m.

Table 3 presents the WW3 namelist parameters used for the ST6 wave physics simulations, with the WW3 ww3_grid setup file showing model implementation of these parameters available in Table S2. In addition to calibrating the wind input to improve the representation of extreme events, we also adjusted the negative wind input parameter (SINA0), following the recommendations of Pathirana *et al*.^37^ based on WW3 test runs compared against Synthetic Aperture Radar (SAR) swell observations across the Pacific Basin. As a result of this adjustment, a slight recalibration of the Discrete Interaction Approximation (DIA) resonance constant (SNL1) was necessary. Furthermore, we adopted a constant decay formulation for swell dissipation (CSTB1 = T). Ice-related cut-offs (CICE0 and CICEN) define thresholds for wave–ice interactions, ensuring appropriate switching between open water and ice-covered dynamics for the simple ice-blocking scheme selected (IC0 in Table 1).

**Table 3:** Specific model parameters for selected physics. Model configuration implementation is available in Table S2 which shows the WW3 ww3_grid setup file.

Parameter	Value	Function	Description
DTIME	20000	PSMC	Swell age for diffusion term (seconds)
CDFAC	1.00	FLX4	Rescaling of drag coefficient (default)
CICE0	0.25	MISC	Ice concentration cut-off (ice-free)
CICEN	0.75	MISC	Ice concentration cut-off (solid ice)
FLAGTR	4	MISC	Flag indicating sea ice obstruction
WCOR1	20	MISC	Apply WCOR2 amplification for winds over threshold (m/s)
WCOR2	0.9	MISC	Amplification factor for winds above WCOR1
SINA0	0.04	SIN6	Factor for negative input from adverse winds
LAMBDA	0.237	SNL1	Discrete interaction approximation (DIA) resonant constant
NLPROP	2.13E + 07	SNL1	DIA proportionality coefficient
SWLB1	0.22E-03	SWL6	Scaling coefficient for swell dissipation
CSTB1	T	SWL6	Swell dissipation with constant decay

**Fig. 4:**
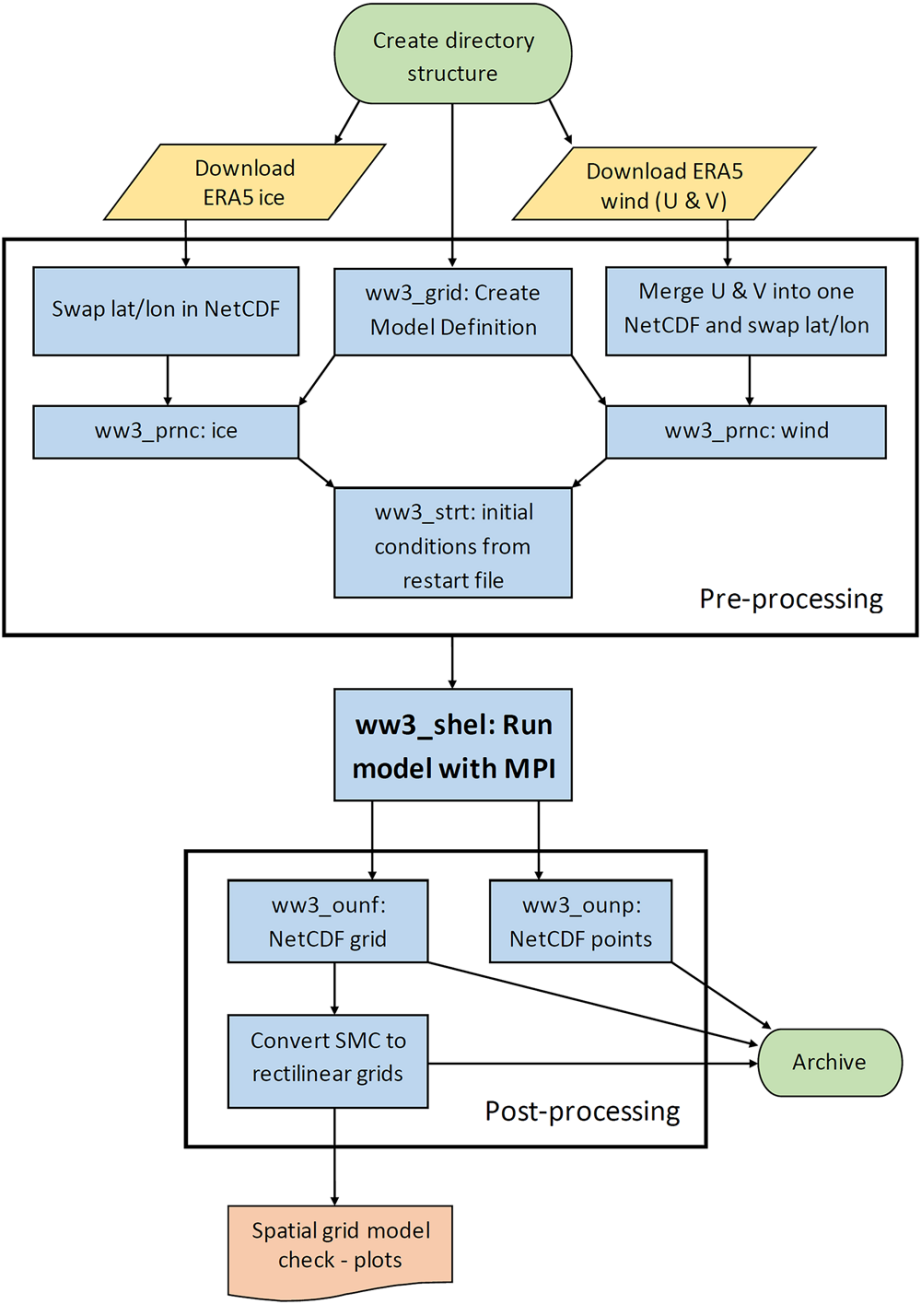
Flowchart illustrating the monthly WAVEWATCH III (WW3) model run process, including data preparation, pre-processing, restart file handling for continuous multi-month simulations, computation, and post-processing steps.

The model runs one calendar month per execution, matching the temporal resolution of the ERA5 input wind and ice data. As illustrated in the flowchart in Fig. 4, each monthly run involves several key stages: initial data preparation, pre-processing, computation, and post-processing. The model operates using a Rose/Cylc suite^38^ that manages the execution order and dependencies.

The ERA5 input data must first be adjusted, including reordering the latitude and longitude, to ensure compatibility with WW3, and then further processed onto the appropriate grid coordinates and units. Restart files, which capture the wave energy state at the final timestep of each previous month, are used to initialise the model state for subsequent runs. To improve efficiency, the model employs Message Passing Interface (MPI) parallelisation, running across 384 processes and reducing computation time to just a few hours per month. Before final archiving, the SMC grid outputs are post-processed and converted into two rectilinear grids: a global grid at 1/8° resolution and an Australasian grid at 1/16° resolution to accommodate users unfamiliar with unstructured grids.

## Data Records

The WHACS dataset is available as bulk wave parameters across the native SMC grid, a global rectilinear grid with 1/8° resolution, a regional Australasian grid with 1/16° resolution (Fig. 1), and full spectral data from a discrete set of selected global data points. The complete set of NetCDF data files from January 1979 to present is published by CSIRO^1^ and indexed within the Research Data Australia with Digital Object Identifier 10.25919/shdk-7p29:

For direct download from a browser via the Data Access Portal: 10.25919/yp77-v026.

Remote access via THREDDS: https://data-cbr.csiro.au/thredds/catalog/catch_all/ACS_WP3_WHACS/ACS_hindcast_DRS/catalog.html.

Available bulk wave parameters are listed in Table 4, and each parameter is stored separately in NetCDF monthly files. The NetCDF files are identified by the filename suffix, with a prefix that denotes the month by start and end date stamps, i.e.

**Table 4:** Bulk wave parameters available for the native SMC grid and the rectilinear regrids (global and Australasia).

WW3 Name	Description	Variable Name	units	SMC Grid	Rectilinear Grids
HS	Significant Height of Wind and Swell Wave	hs	m	✓	✓
WND	Eastward Wind	uwnd	m/s	✓	✓
	Northward Wind	vwnd	m/s	✓	✓
T01	Mean Period (T01)	t01	s	✓	✓
T02	Mean Period (T02)	t02	s	✓	✓
T0M1	Mean Period (T0m1)	t0m1	s	✓	✓
FP	Peak Wave Frequency	fp	Hz	✓	✓
DIR	Mean Wave Direction	dir	degree	✓	✓
DP	Peak Direction	dp		✓	✓
SPR	Directional Spread	spr	degree	✓	
PQP	Goda Peakedness Partition [0–3]	pqp		✓ [0–3]	
PHS	Wave Significant Height Partition [0–3]	phs	m	✓ [0–3]	✓ [0–2]
PTP	Peak Period Partition [0–3]	ptp	s	✓ [0–3]	✓ [0–2]
PDIR	Wave Direction Partition [0–3]	pdir	degree	✓ [0–3]	✓ [0–2]
PDP	Peak Direction Partition [0–3]	pdp	degree	✓ [0–3]	
PPE	JONSWAP Peak Enhancement Factor Partition [0–3]	ppe		✓ [0–3]	
PSPR	Directional Spread Partition [0–3]	pspr	degree	✓ [0–3]	
CGE	Wave Energy Flux	cge	kW/m	✓	✓

< *var* > _WHACS_hindcast_WHACS_ERA5_1hr_ < *yyyymmddhhmm* > - < *yyyymmddhhmm* > .nc

Some corrections were made to the data during post-processing:

- Wave Energy Flux (CgE) attained a negative value of −1 in sea ice regions, and odd negative values in isolated cases due to numerical overflow. These values were reset to NaN or brought within range by changing the datatype from int16 to uint16.
- Peak Wave Frequency (fp) attained negative values at low frequencies close to the minimum spectral frequency, due to a known bug in this version of WW3 (https://github.com/NOAA-EMC/WW3/pull/741). These negative fp values were set to the minimum spectral frequency of the model set up (0.041 Hz).
- Negative values of Goda Peakedness (pqp) partition variables were replaced with the maximum valid value for int16 data format; however, such values would be considered non-physical (https://github.com/NOAA-EMC/WW3/issues/210). Therefore, caution is needed regarding the use of pqp.

The SMC grid output includes partitions 0 to 3 for parameters required to reconstruct the spectrum for locations where WHACS does not have specific spectral output points (see HyWaves^12^). The partitions are produced using the WW3 default spectral partitioning scheme, which groups energy into distinct wave systems rather than relying on a simple separation between sea and swell or fixed frequency cutoffs. This method identifies physically meaningful systems, such as wind seas and multiple swell trains, based on properties like wave age and directional consistency, ensuring a more realistic representation of complex wave fields.

To improve the usability of the dataset, the chunking layout of the SMC data was restructured. The original configuration, which stored data in large spatial blocks across the entire domain (1, 1270610), meaning one time step and 1.27 million spatial points, was suitable for accessing global snapshots at a single time but proved inefficient for extracting time series at individual locations. To address this, the chunking was optimised in both time and space to (372, 11551), significantly enhancing performance for time series analysis. This reconfiguration makes the dataset more efficient in terms of both memory use and processing speed, particularly for climate and oceanographic studies focused on temporal trends and variability. Regridded data at 1/8° (globally) and 1/16° (Australasian region) were also rechunked to improve performance. Metadata was modified to ensure compliance with Climate and Forecast (CF) and Attribute Convention for Data Discovery (ACDD) metadata standards for scientific useability, discoverability, and cataloguing.

The wave spectrum shows increased variability as it approaches intermediate to shallow depths and coastlines. Therefore, to best represent spectral changes for downscaling modelling applications, the coverage of output points should increase with respect to depth and proximity to significant land masses. Decisions regarding coverage of output spectral points in WHACS were made to offer maximum flexibility for a wide range of users, particularly regional modellers who require spectra boundary conditions for coupled hydrodynamic-wave modelling. Domain size and extent need to be optimised for compute, as well as ensuring all necessary processes are captured, with applications ranging from large spatial domains down to focusing on a particular island. Typically, the domain size for coastal water level studies needs to extend further offshore than what is necessary for wave-only downscaling domains to adequately capture the hydrodynamics.

**Fig. 5:**
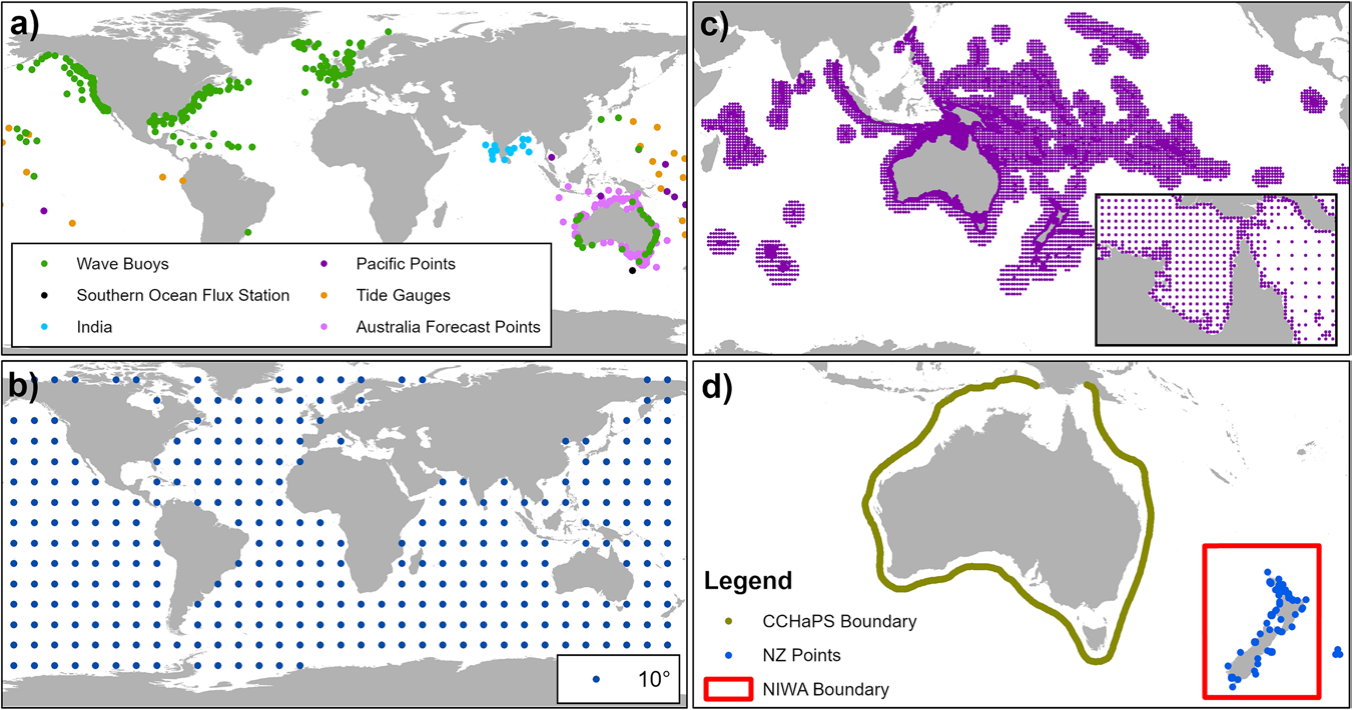
Global spectral output locations (**a**) specific observation and project-based locations, (**b**) regular 10-degree grid, (**c**) Spectral output locations around countries, islands, and shelf regions in the Indo-Pacific, and (**d**) CCHaPS coupled wave-hydrodynamic open boundary around Australia (green), ESNZ boundary (red), and ESNZ requested output locations around New Zealand (blue).

For the Indo-Pacific region, WHACS spectral output points across exclusive economic zones (EEZ) surrounding island nations and territories have a coverage of one degree spacing. For water depths less than 500 metres within EEZs, the coverage increases to 30-minute spacing. Finally, a 25 km buffer region around all major land masses in the ocean basins larger than 1 km^2^ (per USGS Global Islands^39^) has spectral output point coverage with 15 minute spacing (Fig. 5c) that is specific for localised domains.

Figure 5d shows the spectral boundary output around Australia for input into CCHaPS with a spacing of 0.3° (~30 km). Sensitivity tests for other spacing options (~10 km, ~50 km) showed little difference in downscaled wave modelling within the regional domain. Also shown in Fig. 5d is the requested output locations around New Zealand from Earth Sciences New Zealand (ESNZ), and the boundary location (in WW3 spectral boundary format) for their mainland Aotearoa New Zealand wave forecast model^40^.

A limitation of the spectral data output when using SMC grids is that, for each output location, WW3 gives an interpolated value, using the grid locations of the coarser-resolution base grid (1/8° in this case), rather than the higher resolution shelf grid (1/16°). Differences between bulk parameters from the model’s gridded output are occasionally noticeable when compared to manually computed integrated parameters using the spectral data. Regions where this issue is pronounced are usually where there are changes to the bathymetry that are inadequately captured in the WHACS base grid but resolved in the higher resolution grid.

## Technical Validation

The outputs of the WHACS model were validated globally against significant wave height (Hs) derived from 13 satellite altimeters which were calibrated using National Oceanographic Data Centre buoy data^41^. Additionally, for the Australian region, the model’s performance was evaluated by comparing results with a comprehensive dataset of wave buoy observations spanning 1985 to 2020 from the Australian Ocean Data Network (AODN, https://portal.aodn.org.au/)^42^. An improvement in average wave statistics in WHACS as compared to CAWCR hindcast is expected due to the use of ERA5 winds as well as enhancements in the wave model physics and grid settings.

**Fig. 6:**
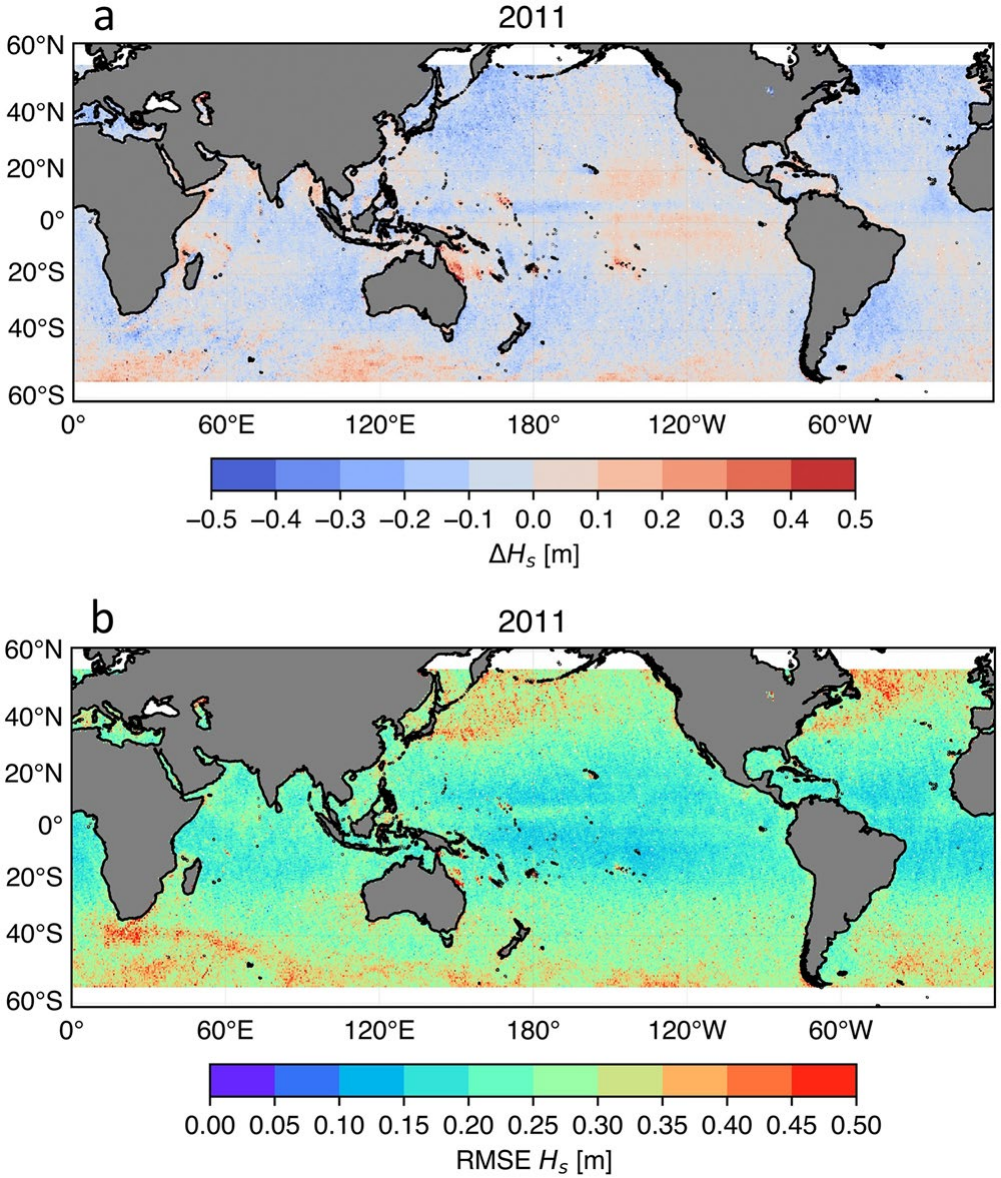
Evaluation of the WHACS significant wave height (Hs) following the chosen ST6 WCORd wind correction calibration using model-altimeter collocations for 2011. Contour plots in the domain 55°S and 55°N excluding sea ice covered areas for the model-altimeter global (**a**) bias (**b**) root mean squared error.

Figure 6a presents the global bias of the WHACS WCORd selected calibration relative to altimeter observations for the year 2011. The overall global bias of the selected WCORd calibration is very low, at −4 cm. However, the model does tend to overestimate significant wave heights in the Southern Ocean. This bias is partly attributed to the exclusion of surface current effects in the wave model^43^. In this region, strong oceanic currents flow in the same direction as the wind-generated waves. When surface currents align with wave propagation, the relative wind speed experienced by the wave field is effectively reduced, leading to lower wave growth than would occur in still water. As a result, neglecting these currents can cause the model to overestimate wave heights. A slight underestimation of the Northern Hemisphere Western Ocean basin may be related to either wind speed underestimation or storm representation issues. Figure 6b shows the global RMSE, with patches of higher RMSE in similar regions to the Southern Ocean bias, but high RMSE values also appear in the northwestern North Pacific and North Atlantic Oceans corresponding with regions of high incidence of extra-tropical cyclone genesis.

**Fig. 7:**
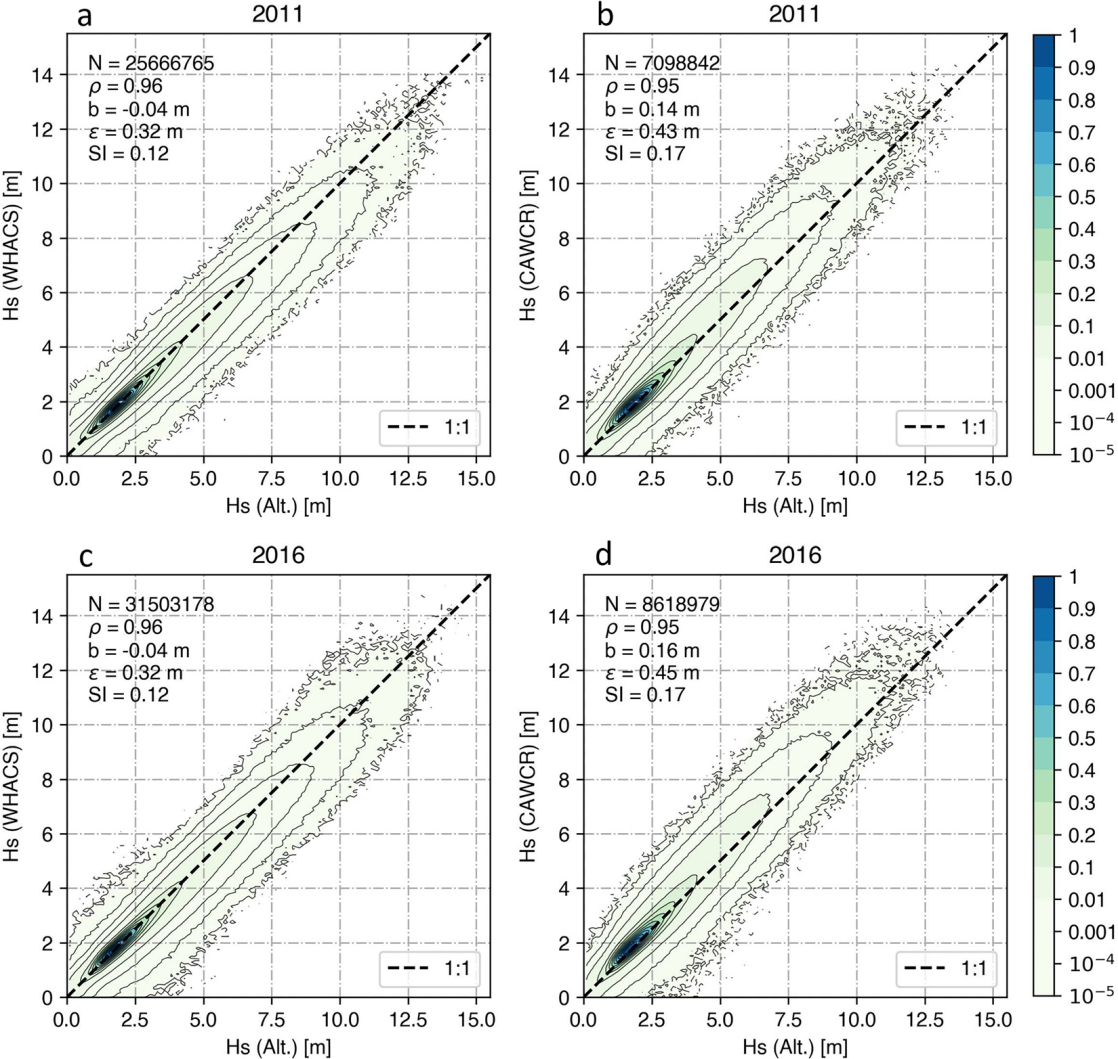
Evaluation of the WHACS significant wave height (Hs) following the chosen ST6 WCORd wind correction calibration using model-altimeter collocations for the years 2011 and 2016. Panels (**a**) to (**d**) show the comparison of the scatter density plots of the model-altimeter Hs collocations from two wave hindcast models in 2011 and 2016, limited to non-ice-infested regions between 55°S and 55°N. Panels (**a**) and (**c**) show co-locations from the WHACS wave hindcast for 2011 and 2016, respectively, while panels (**b**) and (**d**) present co-locations from the CAWCR model for the same years.

Figure 7a–d show the model-altimeter co-location density plots for the years 2011 and 2016 for both the CAWCR and WHACS hindcasts. The CAWCR dataset is divided into two versions: CAWCRv1, which covers the period from 1979 to March 2013, and CAWCRv2, which covers data from April 2013 onwards. The years 2011 and 2016 were selected to account for the differences between the two datasets and to show the consistency of the validation results across different years and satellite missions. The results demonstrate that WHACS improves over the CAWCR wave hindcast in all key statistical metrics when compared to altimeter observations, including higher correlation (ρ), reduced bias (b), lower root mean square error (ε), and a smaller scatter index (SI). Some points on the CAWCR density plots appear slightly higher along the 1:1 line compared to WHACS at the extremes; however, the difference is very subtle. It is not unexpected that CAWCR extremes are well represented, as CFSR winds can overestimate extreme wind speeds^44,45^.

**Fig. 8:**
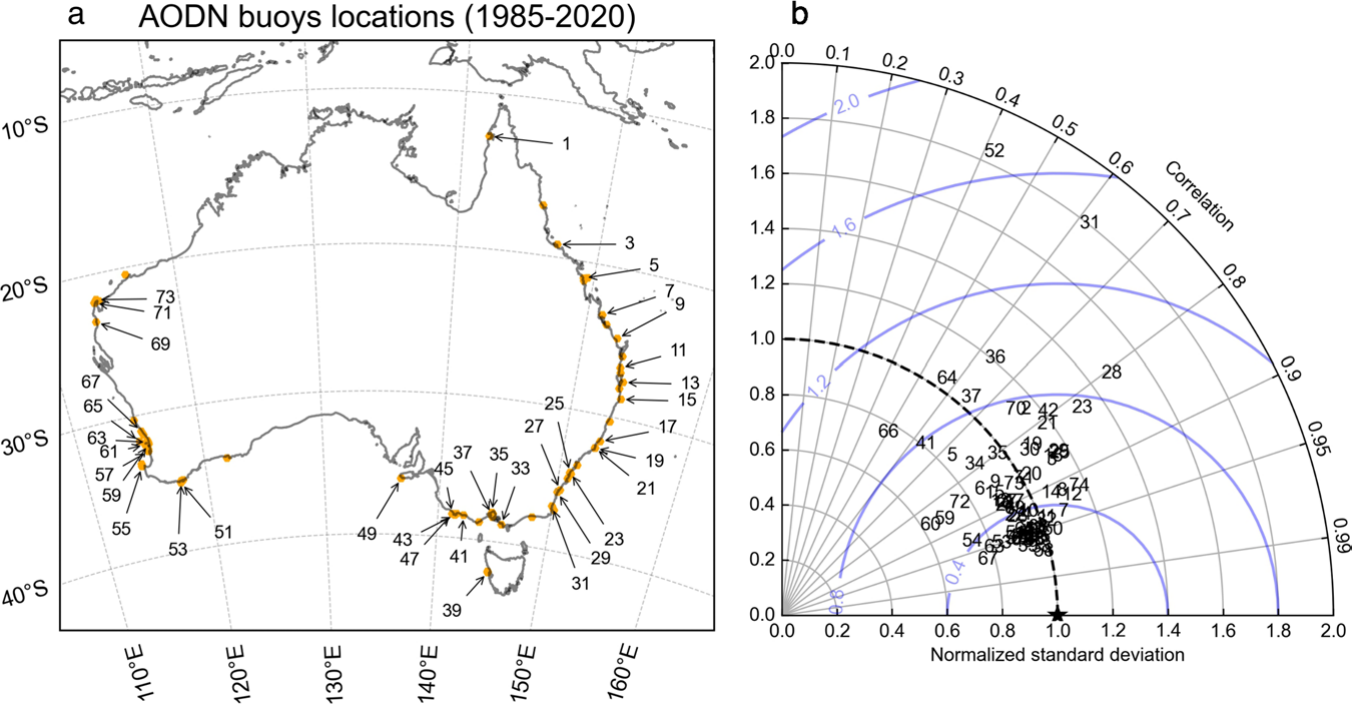
Evaluation of significant wave height (Hs; m) from WHACS against 74 moored wave buoy observations (Table S1) for the period 1985–2020 (source: Australian Ocean Data Network). (**a**) Geographic distribution of AODN moored wave buoys used for buoy/model co-locations. The buoys are located around the Australian continent and are labelled with numeric identifiers corresponding to their entries in Table S1. (**b**) Taylor diagram illustrating the performance of the WHACS model at each buoy location. Each point represents a buoy (labelled with its ID) and shows the normalised standard deviation (radial distance) and the correlation coefficient (angular position) relative to the observed significant wave height (Hs). Concentric blue contours indicate the Root Mean Square Error (RMSE) centred around the point of perfect agreement between the model and observations (black star).

We further assessed the performance of WHACS using a set of *H*_*s*_ observations from AODN moored wave buoys in the Australian continent. The evaluation was conducted by gathering all available measurements from 1985 to 2020 and performing hourly co-locations with the WHACS model outputs. The list of buoys included in this analysis is presented in Table S1. Note that not all available buoys were included, as those located very close to the coast or in extremely shallow waters were excluded due to limitations in the WHACS spatial resolution. AODN wave buoys measure *H*_*s*_ using two different methods: the sea surface significant wave height from time domain analysis (WHTH) and the spectral sea surface significant wave height (WSSH). When both measurements were available for a buoy, all values were considered (obs. ID ‘both’ in Supp. Table 1). The wave buoys are ordered with a numerical ID (num ID) from 1 to 74, starting from the northernmost buoy located in Albatross Bay, and moving in a clockwise direction around the Australian continent, as shown in Fig. 8a.

Figure 8b shows the Taylor plot of the 1985–2020 hourly model-buoy collocations^46^. Each point represents a buoy (labelled with its numerical ID) and shows the normalised standard deviation (radial distance) and the correlation coefficient (angular position) relative to the observed Hs. Concentric blue contours indicate the Root Mean Square Error (RMSE) centred around the point of perfect agreement between the model and observations (black star). The WHACS model underperforms at the Merimbula (31) and Albany 02 (52) buoy locations, as the model-to-buoy comparison struggles to match values close to these buoys, most likely due to their inshore coastal locations. Overall, however, the global wave hindcast performs remarkably well in coastal areas, especially considering that it is a global modelling effort and is not focused on resolving fine-scale (below 1 km resolution) topography. These results further highlight the value of the variable-resolution SMC grid approach.

**Fig. 9:**
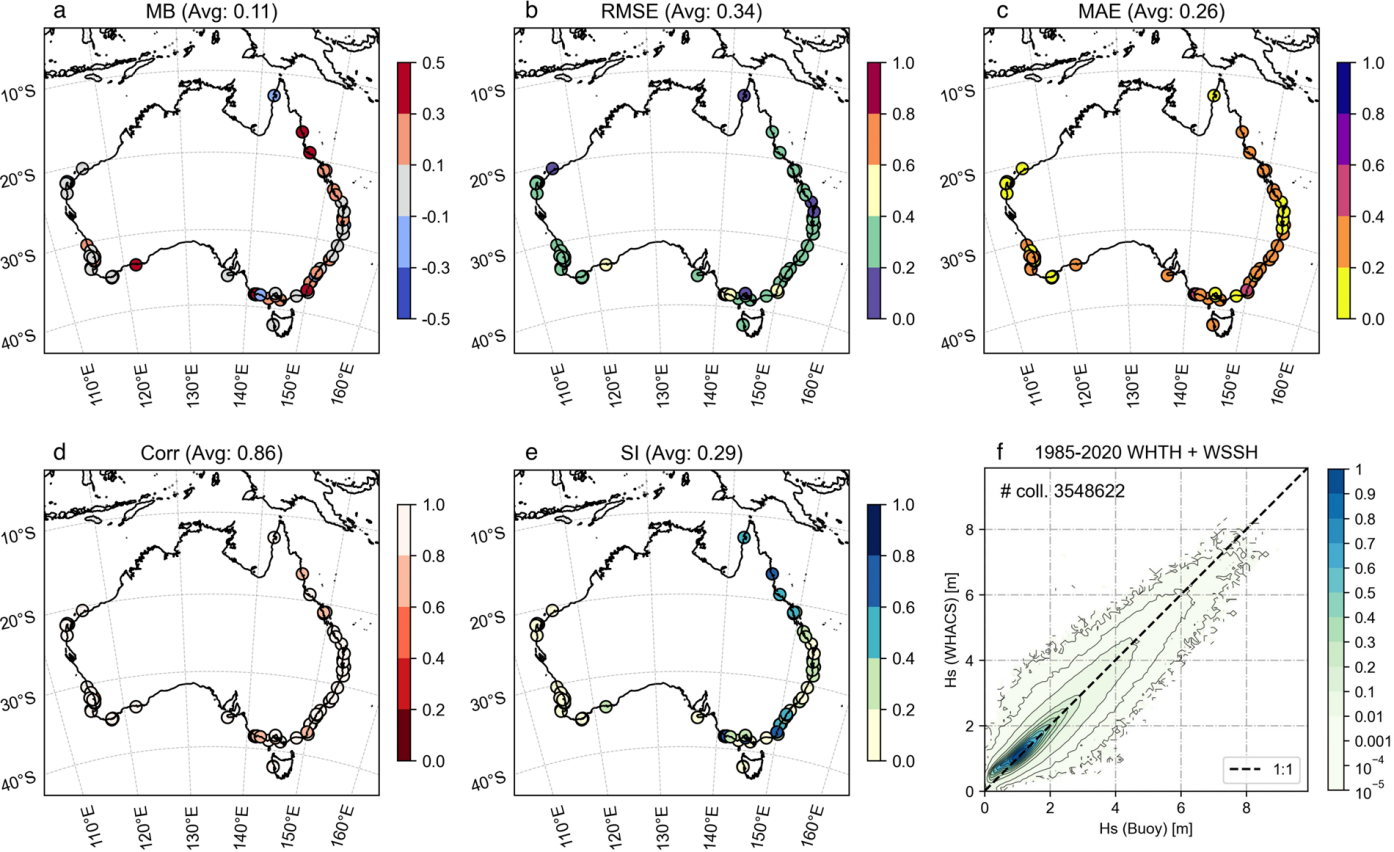
Evaluation of the WHACS wave model’s significant wave height (Hs) against wave height observations from 74 moored buoys (Table S1) for the period 1985–2020 (source: Australian Ocean Data Network). Subplots (**a**–**e**) show scatter plots of model versus observed Hs for locations around the Australian continent (MB: Mean Bias, RMSE: Root Mean Square Error, MAE: Mean Absolute Error, Corr: Pearson’s Correlation, SI: Scatter Index) (**f**) Density plot summarizing the buoy/model collocations performance across the entire buoy dataset.

Figure 9a–e present spatial scatterplots of key statistical metrics for *H*_*s*_: Mean Bias (MB), Mean Absolute Error (MAE), Pearson’s Correlation (Corr), and SI, at the 74 moored buoy locations listed in Table S1. The coloured dots show that the agreement between the wave model and the buoy observations is poorer in the New South Wales and Queensland regions, with higher bias, RMSE, and Scatter Index values. Figure 6 indicates that the Great Barrier Reef and Coral Sea exhibit some of the largest discrepancies. According to Dong *et al*.^47,48^, such errors across the GBR arise from the underestimation of small-scale reef energy dissipation which can be improved with the application of subgrid parameterization. However the bias extends further into the Coral Sea, far beyond the reef, indicating the issue is associated with how well the atmospheric model captures the dynamics of the region.

Figure 9f displays the density plot of over 3.5 million hourly buoy–model co-locations for all 74 buoys. The highest density of collocations aligns closely with the 1:1 line, indicating strong overall performance of the WHACS dataset. A slight positive bias is observed for *H*_*s*_ below one metre, suggesting some underperformance of WHACS in low-energy conditions.

**Fig. 10:**
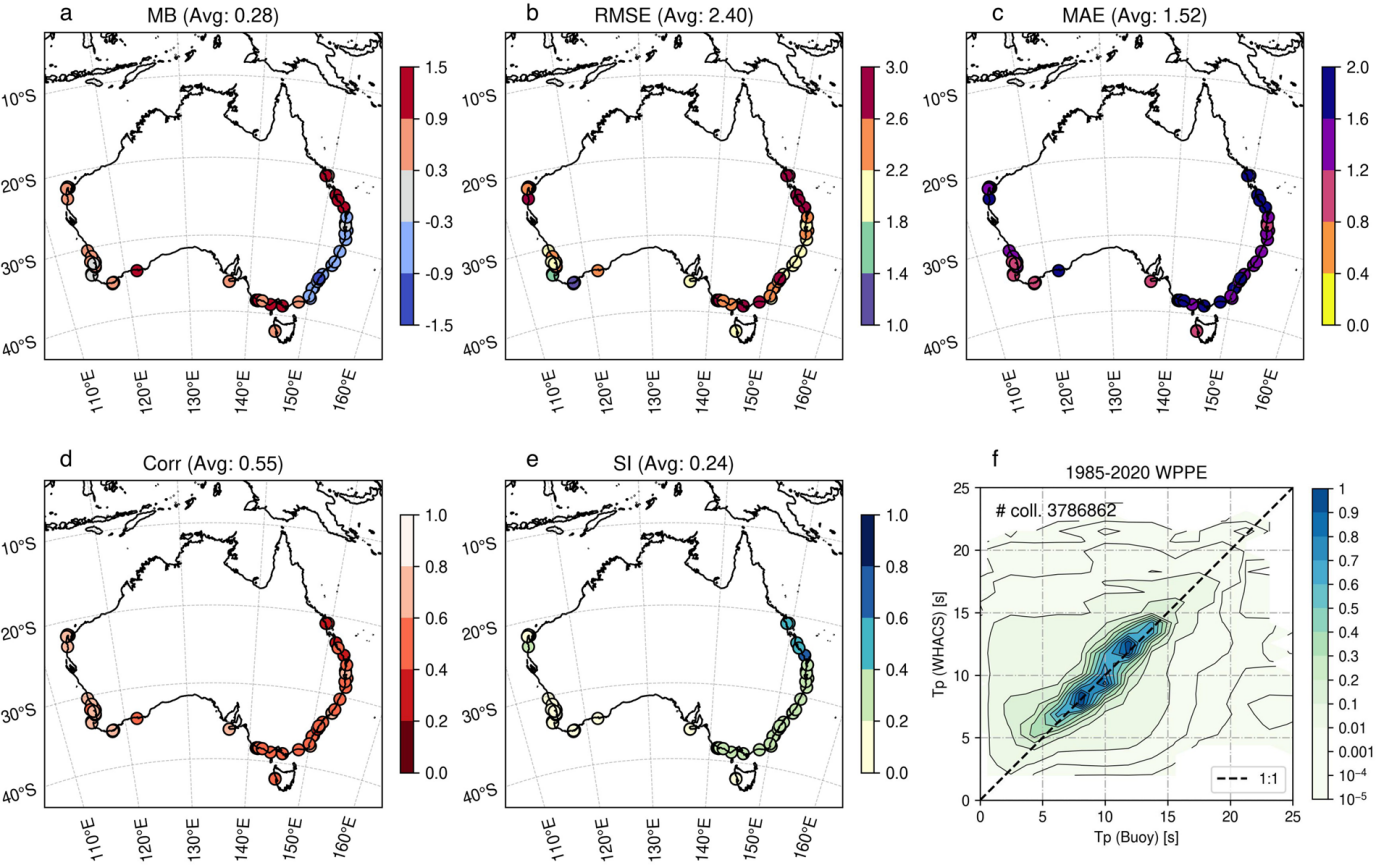
Evaluation of the WHACS wave model’s peak period (Tp) against wave height observations from 74 moored buoys (Table S1) for the period 1985–2020 (source: Australian Ocean Data Network). Subplots (**a**–**e**) show scatter plots of model versus observed Tp for locations around the Australian continent (MB: Mean Bias, RMSE: Root Mean Square Error, MAE: Mean Absolute Error, Corr: Pearson’s Correlation, SI: Scatter Index) (**f**) Density plot summarizing the buoy/model collocations performance across the entire buoy dataset.

Figure 10a–e present spatial scatterplots similar to Fig. 9 but for *Tp*. The mean bias is small and positive, but various regionally, with negative values along the New South Wales coast and into southern Queensland, and positive further north along the Queensland coast and much of the rest of the country (with some neutral bias on the west coast of Western Australia). Error metrics show larger errors at exposed east/southeast sites and generally smaller errors along parts of the west coast. The correlation is moderate (0.55), indicating the model captures broad variability in *Tp* but misses some event-scale details. The scatter index suggests relative errors on the order of a quarter of the mean *Tp*, increasing at a few east/south locations.

The density plot (Fig. 10f) shows *Tp* clustered along the 1:1 line for ~8–12 s, with increasing spread at the tails. There’s a tendency to underpredict longer periods (>~14 s) (points falling below the 1:1 line) and a hint of overprediction for short periods (<~6 s). Results are largely comparable to Dong *et al*.^47^ which also showed positive/negative bias in similar regions and the highest RMSE values in southern Queensland. Overall, WHACS provides a reasonably unbiased depiction of *Tp* with moderate skill.

### Case Study: April 2021 Extra-Tropical Storm

**Fig. 11:**
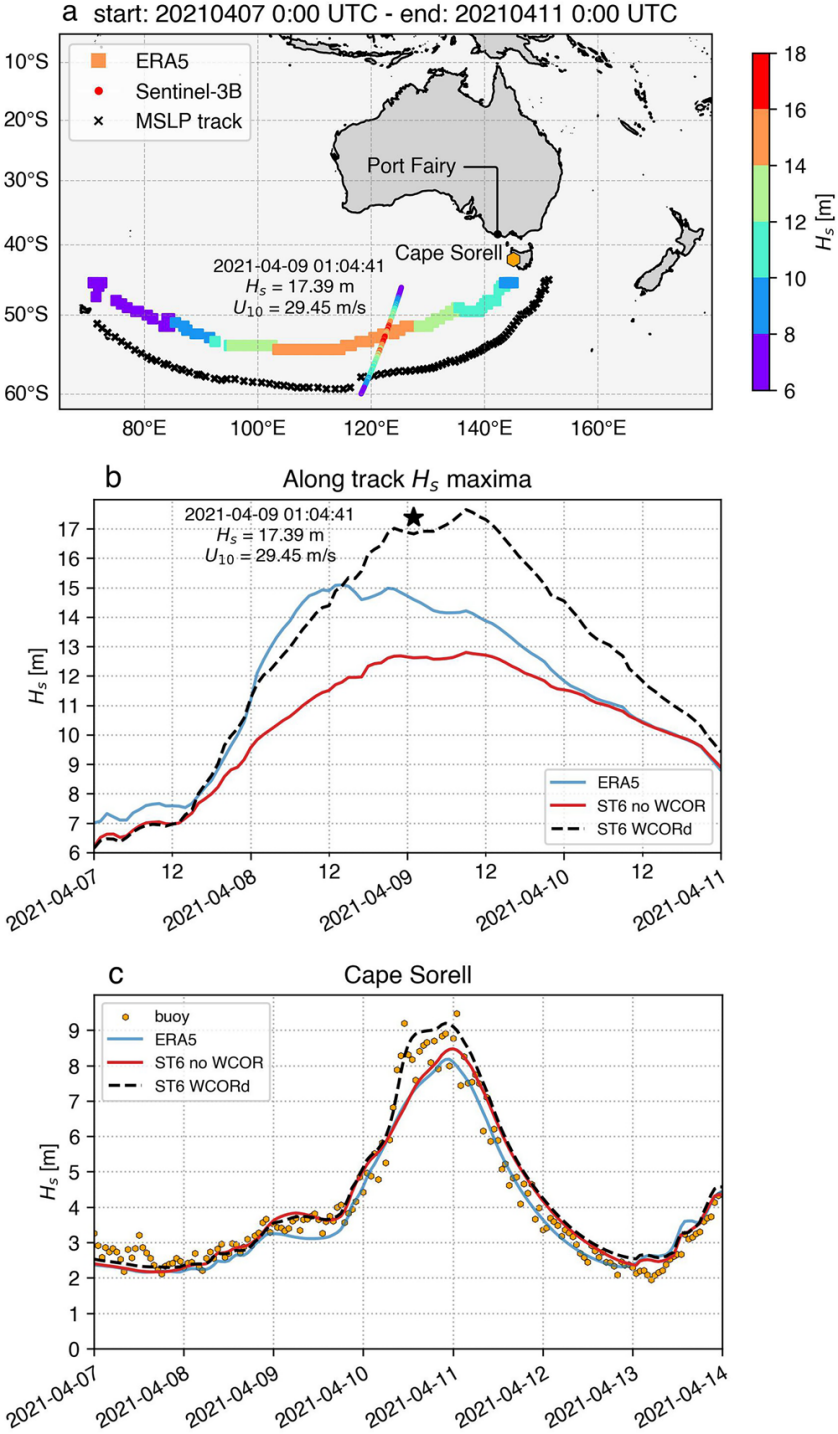
(**a**) Map showing the track and wave height development of the April 2021 extra-tropical storm, which originated over the Indian Ocean and made landfall on the west coast of Tasmania. The storm track is represented by the ERA5 Minimum Mean Sea Level Pressure (MSLP) values, shown as black crosses. The coloured square scatter plot illustrates the simulated maximum significant wave heights (Hs) by ERA5, highlighting the intensification of the storm and the trajectory of Hs maxima on the left side of the storm centre, as is typical in the Southern Hemisphere clockwise rotating storms. The coloured circle scatter plot shows the values observed by the Sentinel-3B satellite on the night of April 9th, 2021, with a maximum observed Hs of 17.39 m at 01:04:41 UTC. (**b**) Maximum Hs at each hourly time step along the storm track, demonstrating the evolution of wave energy as the storm progressed. The black star corresponds to the maximum Hs recorded by Sentinel-3B, also shown in (**a**). (**c**) Comparison of the Cape Sorell buoy measurements and wave model simulations of Hs time series. The Cape Sorell buoy, located approximately 10 km offshore from the west coast of Tasmania, is marked as an orange dot in (**a**).

A recent example of an extreme coastal hazard event with significant impacts occurred in April 2021 along the southwest coast of Victoria, particularly around Port Fairy (see Fig. 11a), from large swell waves which propagated from a storm that developed in the southern Indian Ocean. The powerful swell displaced substantial rocks and boulders from the seawall along the South Beach area, moving them inland by 20 to 30 m. This event raised concerns among local residents and authorities about the potential effects of climate change and sea-level rise on coastal infrastructure^49^.

The highest Hs recorded for this event was 17.39 m, as observed from a Sentinel-3B satellite altimeter passing in the centre of the storm (Fig. 11a). Meucci *et al*.^50^ investigated the performance of spectral wave models in simulating the April 2021 Extra-Tropical storm. In this study, we revisited their work to assess the improvements made with the current ERA5 wind speed calibration (WCORd) in simulating the Hs along the storm’s entire track, as well as at the coastal impact point. As shown in Fig. 11b,c, ERA5 significantly underestimated wave heights, with a maximum Hs of 14.59 m at the location and time of the Sentinel-3B measurement. The standard ERA5-driven WW3 ST6 simulation, without wind calibration, predicted a maximum Hs of 12.62 m at the same location and time. However, a notable improvement is observed when the ERA5 wind speed calibration is applied in the current ST6 model (ST6 WCORd; Table 2), which simulates an Hs of 16.83 m (Fig. 11b), closer to the value recorded by the Sentinel-3B altimeter (black star).

Additionally, extending this comparison to the storm’s impact point on the west of Tasmania, we compared the model results with observations from the Cape Sorell wave buoy (Fig. 11a). Figure 11c presents a comparison of ERA5 and the two ST6 model runs against the buoy measurements (orange dots). Improvements in representing the energy in the storm region led to a better simulation of the swell waves impacting the West Coast of Tasmania. Notably, the WHACS ST6 WCORd simulation (black dashed line) successfully captured both swell peaks observed by the Cape Sorell buoy. Both the buoy measurements and the ST6 WCORd simulation show that a swell system first impacted the region prior to the arrival of the storm, followed by the arrival of a second, larger wave system at the end of 10th of April, coinciding with the storm’s arrival.

## Supplementary information

Table S1 and Table S2

## Data Availability

The WHACS dataset is available as bulk wave parameters across the native SMC grid, a global rectilinear grid with 1/8° resolution, a regional Australasian grid with 1/16° resolution, and full spectral data from a discrete set of selected global data points. The complete set of NetCDF data files from January 1979 to present is published by CSIRO and indexed within the Research Data Australia with Digital Object Identifier 10.25919/shdk-7p29: For direct download from a browser via the Data Access Portal: 10.25919/yp77-v026 Remote access via THREDDS: https://data-cbr.csiro.au/thredds/catalog/catch_all/ACS_WP3_WHACS/ACS_hindcast_DRS/catalog.html Bulk wave parameters are stored separately in NetCDF monthly files. The NetCDF files are identified by the filename suffix, with a prefix that denotes the month by start and end date stamps, i.e., < *var* > _WHACS_hindcast_WHACS_ERA5_1hr_ < *yyyymmddhhmm* > - < *yyyymmddhhmm* > .nc

## Code availability

A notebook has been made available that forms a basic guide in using data produced from WHACS, with examples of a number of basic tasks that may be undertaken by researchers and data users, and tricks and tips to ensure smooth and efficient use of the data as supplied by the CSIRO Data Access Portal (available as a supporting file in the CSIRO Data Access Portal: whacs_examples.ipynb).

## References

[1] Smith, G. *et al*. WHACS: Wave Hindcast for the Australian Climate Service. at, 10.25919/83kj-kp95 (2024).

[2] Smith, G. A. *et al*. Global wave hindcast with Australian and Pacific Island Focus: From past to present. *Geosci. Data J*. **8** (2021).

[3] Durrant, T., Greenslade, D. J. M., Hemer, M. & Trenham, C. *A Global Wave Hindcast Focussed on the Central and South Pacific*. *CAWCR Technical Report No. 070* http://www.cawcr.gov.au/technical-reports/CTR_070.pdf (2014).

[4] Leach, C. *et al*. Measuring drivers of shoreline and subaerial beach change using limited datasets in a temperate, wave-dominated sandy system: Inverloch, Australia. *Ocean Coast. Manag*. **240** (2023).

[5] Ibaceta, R. & Harley, M. D. Data-driven modelling of coastal storm erosion for real-time forecasting at a wave-dominated embayed beach. *Coast. Eng.* **193**, 104596 (2024).

[6] Sun, Z. *et al*. Study on the Wind and Wave Environmental Conditions of the Xisha Islands in the South China Sea. *J. Mar. Sci. Eng*. **10** (2022).

[7] Hemer, M. A. *et al*. A revised assessment of Australia’s national wave energy resource. *Renew. Energy*, 10.1016/j.renene.2016.08.039 (2017).

[8] Gao, Q. *et al*. Assessment of wind and wave power characteristic and potential for hybrid exploration in Australia. *Renew. Sustain. Energy Rev*. **168** (2022).

[9] Barnes, A. T. *et al*. Rising sea levels and the increase of shoreline wave energy at American Samoa. *Sci. Rep*. **14** (2024). 10.1038/s41598-024-55636-y PMC10908797 38431739

[10] Morim, J. *et al*. A global ensemble of ocean wave climate statistics from contemporary wave reanalysis and hindcasts. *Sci. Data* **9** (2022).

[11] Wandres, M., Espejo, A. & Damlamian, H. Wave Climate Variability and Trends in Tuvalu Based on a 44-Year High-Resolution Wave Hindcast. *J. Geophys. Res. Ocean*. **128** (2023).

[12] Ricondo, A. *et al*. HyWaves: Hybrid downscaling of multimodal wave spectra to nearshore areas. *Ocean Model*. **184** (2023).

[13] Espejo, A. *et al*. Efficient coastal inundation early-warning system for low-lying atolls, dealing with lagoon and ocean side inundation in Tarawa, Kiribati. *Weather Clim. Extrem*. **42** (2023).

[14] Wong, P. P. *et al*. Coastal systems and low-lying areas. in 361–409, 10.1017/CBO9781107415379.010 (2014).

[15] Hernaman, V. *et al*. The Australian Climate Service Coupled Hydrodynamic–Wave Coastal Hazards Prediction System (CCHaPS): Development, Implementation and Validation (2025).

[16] Zieger, S. The Australian wave forecast system with statistical wind correction. *J. South. Hemisph. Earth Syst. Sci*. **75** (2025).

[17] Tolman, H. L., Banner, M. L. & Kaihatu, J. M. The NOPP operational wave model improvement project. *Ocean Model*. **70** (2013).

[18] Ardhuin, F., Chapron, B. & Collard, F. Observation of swell dissipation across oceans. *Geophys. Res. Lett*. **36** (2009).

[19] Zieger, S., Babanin, A. V., Erick Rogers, W. & Young, I. R. Observation-based source terms in the third-generation wave model WAVEWATCH. *Ocean Model*, 10.1016/j.ocemod.2015.07.014 (2015).

[20] WW3DG, T. W. I. D. G. User manual and system documentation of WAVEWATCH III version 6.07. *NOAA / NWS / NCEP / MMAB Tech. Note* (2019).

[21] Liu, Q. *et al*. Observation-based source terms in the third-generation wave model WAVEWATCH III: Updates and verification. *J. Phys. Oceanogr*. 10.1175/JPO-D-18-0137.1 (2019).

[22] Su, C.H. *et al*. *BARRA2: Development of the next-Generation Australian Regional Atmospheric Reanalysis*. (2022).

[23] Hersbach, H. *et al*. The ERA5 global reanalysis. *Q. J. R. Meteorol. Soc*. **146** (2020).

[24] Fan, W. *et al*. Evaluation of global reanalysis land surface wind speed trends to support wind energy development using *in situ* observations. *J. Appl. Meteorol. Climatol*. **60** (2020).

[25] Liu, Q. *et al*. Global Wave Hindcasts Using the Observation-Based Source Terms: Description and Validation. *J. Adv. Model. Earth Syst*. **13** (2021).

[26] Tolman, H. L. Treatment of unresolved islands and ice in wind wave models. *Ocean Model.* **5**, 219–231 (2003).

[27] Wedd, R. *et al*. ACCESS-S2: the upgraded Bureau of Meteorology multi-week to seasonal prediction system. *J. South. Hemisph. Earth Syst. Sci*. **72** (2022).

[28] Zieger, S. & Greenslade, D. J. M. *A Multiple-Resolution Global Wave Model – AUSWAVE-G3*. *Bureau Research Report* vol. 051 (Bureau of Meteorology, 2021).

[29] Rogers, E. W., Babanin, A. V. & Wang, D. W. Observation-consistent input and whitecapping dissipation in a model for wind-generated surface waves: Description and simple calculations. *J. Atmos. Ocean. Technol*. **29** (2012).

[30] Valiente, N. G. *et al*. The impact of wave model source terms and coupling strategies to rapidly developing waves across the north‐west european shelf during extreme events. *J. Mar. Sci. Eng*. **9** (2021).

[31] Soran, M. B., Amarouche, K. & Akpınar, A. Spatial calibration of WAVEWATCH III model against satellite observations using different input and dissipation parameterizations in the Black Sea. *Ocean Eng*. **257** (2022).

[32] Gandoin, R. & Garza, J. Underestimation of strong wind speeds offshore in ERA5: evidence, discussion and correction. *Wind Energy Sci.* **9**, 1727–1745 (2024).

[33] Dodet, G. *et al*. The Sea State CCI dataset v1: Towards a sea state climate data record based on satellite observations. *Earth Syst. Sci. Data* **12** (2020).

[34] Alday, M., Accensi, M., Ardhuin, F. & Dodet, G. A global wave parameter database for geophysical applications. Part 3: Improved forcing and spectral resolution. *Ocean Model*. **166** (2021).

[35] Elshinnawy, A. I., Menéndez, M. & Medina, R. A parameterization for the correction of ERA5 severe winds for extreme ocean wave modelling. *Ocean Eng.* **312**, 119048 (2024).

[36] Ricciardulli, L. & Wentz, F. J. *Remote Sensing Systems ASCAT C-2015 Daily Ocean Vector Winds on 0.25 Deg Grid, Version 02.1*. *Santa Rosa, CA*: www.remss.com (2016).

[37] Pathirana, S., Young, I. & Meucci, A. Modelling swell propagation across the Pacific. *Front. Mar. Sci*. **10** (2023).

[38] Oliver, H. *et al*. Workflow Automation for Cycling Systems. *Comput. Sci. Eng*. 10.1109/MCSE.2019.2906593 (2019).

[39] Sayre, R. *et al*. A new 30 meter resolution global shoreline vector and associated global islands database for the development of standardized ecological coastal units. *J. Oper. Oceanogr*. **12** (2019).

[40] Santana, R. *et al*. Wave forecast investigations on downscaling, source terms, and tides for Aotearoa New Zealand. *Geosci. Model Dev. Discuss.* **2024**, 1–35 (2024).

[41] Ribal, A. & Young, I. R. 33 years of globally calibrated wave height and wind speed data based on altimeter observations. *Sci. data*, 10.1038/s41597-019-0083-9 (2019). 10.1038/s41597-019-0083-9 PMC6541622 31142742

[42] Integrated Marine Observing System (IMOS). *Wave Buoys Observations - Australia - Delayed (National Wave Archive)*. https://catalogue-imos.aodn.org.au/geonetwork/srv/eng/catalog.search#/metadata/2807f3aa-4db0-4924-b64b-354ae8c10b58 (2023).

[43] Rapizo, H., Durrant, T. H. & Babanin, A. V. An assessment of the impact of surface currents on wave modeling in the Southern Ocean. *Ocean Dyn*. **68** (2018).

[44] Timmermans, B., Patricola, C. & Wehner, M. Simulation and analysis of extremehurricane-drivenwave climate under two ocean warming scenarios. *Oceanography* **31** (2018).

[45] Sharmar, V. D., Markina, M. Y. & Gulev, S. K. Global Ocean Wind-Wave Model Hindcasts Forced by Different Reanalyzes: A Comparative Assessment. *J. Geophys. Res. Ocean*. **126** (2021).

[46] Taylor, K. E. Summarizing multiple aspects of model performance in a single diagram. *J. Geophys. Res. Atmos*. **106** (2001).

[47] Dong, X. *et al*. Numerical simulations of ocean surface waves along the Australian coast with a focus on the Great Barrier Reef. *Geosci. Model Dev*. **18** (2025).

[48] Dong, X. *et al*. A 45-year high-resolution unstructured wave hindcast for the Australian coast: Validation and climatological insights. *Coast. Eng*. **204** (2025).

[49] ABC. Big surf flips boulders across road in Port Fairy, reigniting climate change, coastal erosion concerns. *Australian Broadcasting Corporation News* https://www.abc.net.au/news/2021-04-12/port-fairy-big-surf-damage-sea-level-rise/100063670 (2021).

[50] Meucci, A. *et al*. Evaluation of Spectral Wave Models Physics as Applied to a 100-Year Southern Hemisphere Extra-Tropical Cyclone Sea State. *J. Geophys. Res. Ocean*. **128** (2023).

[51] Cavaleri, L. & Rizzoli, P. M. Wind wave prediction in shallow water: Theory and applications. *J. Geophys. Res. Ocean.* **86**(C11) 10961–10973, 10.1029/JC086iC11p10961 (1981).

[52] Babanin, A. Breaking and Dissipation of Ocean Surface Waves. Breaking and Dissipation of Ocean Surface Waves 10.1017/CBO9780511736162 (2011).

[53] Hwang, P. A. A Note on the Ocean Surface Roughness Spectrum. *J. Atmos. Ocean. Technol.* **28**(3) 436–443, 10.1175/2010JTECHO812.1 (2011).

[54] Miche, A. Mouvements ondulatoire de la mer en profondeur croissante ou décroissante. Première partie. Mouvements ondulatoires périodiques et cylindriques en profondeur constante. *Ann. des Ponts Chaussés* (1944).

[55] Hasselmann, S., Hasselmann, K, Allender, J. H. & Barnett, T. P. Computations and Parameterizations of the Nonlinear Energy Transfer in a Gravity-Wave Spectrum. Part II: Parameterizations of the Nonlinear Energy Transfer for Application in Wave Models. *J. Phys. Oceanogr.* **15**(11) 1378–1391, 10.1175/1520-0485(1985)015 (1985).

[56] Ardhuin, F., O’Reilly, W. C., Herbers, T. H. C. & Jessen, P. F. Swell transformation across the continental shelf. Part I: Attenuation and directional broadening. *J. Phys. Oceanogr*. **33** (2003).

[57] Battjes, J. A. & Janssen, J. P. F. M. Energy Loss and Set-up Due to Breaking of Random Waves. *Proc. Coast. Eng. Conf.* **16** (32) 10.9753/icce.v16.32 (1979).

[58] Young, I. R. & Ribal, A. Can Multi-Mission Altimeter Datasets Accurately Measure Long-Term Trends in Wave Height? *Remote Sens*. **14** (2022).

