# Supplementary material for: WHACS: An Improved Global Wave Hindcast for the Australian Climate Service
Source: Sci Data. 2026 Feb 21;13:558. doi: 10.1038/s41597-026-06864-6 (PMC13066415; doi:10.1038/s41597-026-06864-6)
Supplement: Supplementary file 1 — Table S1 and Table S2 [file 41597_2026_6864_MOESM1_ESM.pdf]

Table S1. List of AODN wave buoys used for the 36-year (1985–2020) hourly Significant Wave Height (Hs) buoy/model collocations. The list of AODN buoys is arranged in a clockwise direction around the Australian continent, starting from the northernmost buoy located in Albatross Bay (see also Figure 7a).

num ID	Site	Latitude (°)	Longitude (°)	Depth (m)	obs. ID	# Coll.
1	Albatross Bay	-12.69	141.68	13.87	WHTH	16243
2	Cairns	-16.73	145.72	17.58	WHTH	111064
3	Townsville	-19.16	147.06	21.08	WHTH	68340
4	Mackay	-21.04	149.55	40.23	WHTH	98803
5	Mackay Inner	-21.1	149.26	17.49	WHTH	14205
6	Hay Point	-21.27	149.31	16.12	WHTH	45761
7	Emu Park	-23.31	151.07	23.17	WHTH	66308
8	Gladstone	-23.9	151.5	17.11	WHTH	50509
9	Bundaberg	-24.67	152.5	21.66	WHTH	1913
10	Wide Bay	-25.8	153.17	45.27	WHTH	9691
11	Mooloolaba	-26.57	153.18	35.05	WHTH	27740
12	North Moreton Bay	-26.9	153.28	35.59	WHTH	16035
13	Brisbane	-27.49	153.63	78.33	WHTH	68876
14	Gold Coast	-27.97	153.44	18.89	WHTH	54417
15	Byron Bay	-28.66	153.75	77.45	WHTH	219076
16	Coffs Harbour	-30.34	153.28	71.25	WHTH	236839
17	Crowdy Head	-31.83	152.85	73.35	WHTH	233108
18	BoomerangB	-32.35	152.56	34.55	both	3840
19	BoomerangB2	-32.35	152.56	34.55	both	7720
20	BoomerangA2	-32.34	152.55	18.26	both	14448
21	BoomerangA	-32.34	152.55	8.09	both	3612
22	Sydney	-33.77	151.4	80.64	WHTH	187892
23	Woonona	-34.35	150.93	18.18	both	7052
24	Port Kembla	-34.47	151.02	86.23	WHTH	237870
25	FairyMeadow	-34.4	150.91	19.18	both	5478
26	Gerroa	-34.81	150.81	32.56	both	1828
27	Batemans Bay	-35.71	150.36	72.46	WHTH	236075
28	Broulee	-35.85	150.19	13.63	both	2500
29	Bengello	-35.88	150.16	10.6	both	2498
30	Eden	-37.18	150.16	92.2	WHTH	238647
31	Merimbula	-36.91	149.92	14.83	both	2210
32	Lakes-entrance	-37.92	148.35	51.27	WSSH	3874
33	Inverloch	-38.75	145.75	50.78	WSSH	8743
34	Ppb-central	-38.06	144.87	25.12	WSSH	542
35	Werribee	-37.98	144.78	13.74	WSSH	530
36	Rosebud	-38.3	144.87	17.3	WSSH	469
37	Indented-head	-38.14	144.75	14.79	WSSH	537
38	Cape Sorell 01	-42.12	145.03	100	WHTH	161663

39	Cape Sorell 02	-42.2	145.05	106.36	both	32350
40	Apollo-bay	-38.75	143.72	31.96	WSSH	8079
41	Port-fairy	-38.38	142.29	28.84	WSSH	7823
42	Dutton-way	-38.32	141.66	20.38	WSSH	4498
43	Cape-nelson	-38.42	141.51	31.48	WSSH	5576
44	Cape-bridgewater-dc	-38.36	141.28	70.47	WSSH	3323
45	Cape-bridgewater-fb	-38.36	141.28	65.32	WSSH	3326
46	Cape-bridgewater	-38.36	141.27	65.32	WSSH	4799
47	Cape-bridgewater-sc	-38.36	141.27	65.32	WSSH	3326
48	Cape Du Couedic 01	-36.07	136.62	69.28	WHTH	135527
49	Cape Du Couedic 02	-36.07	136.62	69.28	both	34366
50	Esperance 04	-34	121.9	54.37	WSSH	112643
51	Albany 04	-35.2	117.72	66.24	WSSH	124757
52	Albany 02	-35.03	117.94	15.38	WSSH	4373
53	TORBAY-EAST	-35.07	117.78	28.43	WSSH	3222
54	TORBAY-WEST	-35.07	117.77	28.43	WSSH	1512
55	Cape Naturaliste 02	-33.53	114.76	52.22	WSSH	138251
56	Cape Naturaliste 01	-33.36	114.78	56.25	WSSH	40705
57	Dawesville 01	-32.6	115.55	27.51	WSSH	5902
58	Mandurah	-32.45	115.57	31.78	WSSH	47188
59	Cockburn	-32.11	115.69	16.91	WSSH	55191
60	Dawesville 03	-32.1	115.63	19.32	WSSH	4818
61	Rottnest Island 01	-32.11	115.4	46.51	WSSH	46374
62	Rottnest Island 02	-32.09	115.41	47.21	WSSH	138272
63	Cottesloe	-31.98	115.69	18.83	WSSH	170963
64	HILARYS	-31.85	115.65	31.38	WSSH	1058
65	PERTH-CANYON	-31.79	115.02	306.47	WSSH	1813
66	Guilderton 01	-31.4	115.43	33.21	WSSH	1897
67	Ledge Point	-31.13	115.31	29.47	WSSH	19076
68	Jurien Bay 02	-30.29	114.91	42.81	WSSH	176154
69	Coral Bay	-23.17	113.74	19.73	WSSH	2110
70	TANTABIDDI	-21.9	113.93	34.32	WSSH	1388
71	Tantabiddi	-21.89	113.93	34.32	WSSH	11486
72	EXMOUTH-GULF	-21.84	114.28	23	WSSH	1373
73	Exmouth	-21.7	114.1	53.57	WSSH	24456
74	DAMPIER	-20.48	116.52	22.28	WSSH	881

Table S2. Wavewatch III grid preprocessor setup files to create the binary file mod_def.ww3 that describes the model domain, spectral discretization, and configuration.

ww3_grid.nml

```
&SPECTRUM_NML
  SPECTRUM%XFR       =  1.10          ! frequency increment
  SPECTRUM%FREQ1     =  0.04118        ! first frequency (Hz)
  SPECTRUM%NK        =  28            ! number of frequencies (wave
  SPECTRUM%NTH       =  30            ! number of direction bins
  SPECTRUM%THOFF     = 0.5             ! relative offset of first di
/
&RUN_NML
  RUN%FLDRY = F           ! dry run (I/O only, no calculation)
  RUN%FLCX  = T          ! x-component of propagation
  RUN%FLCY  = T          ! y-component of propagation
  RUN%FLCTH = T           ! direction shift
  RUN%FLCK  = F          ! wavenumber shift
  RUN%FLSOU = T           ! source terms
/
&TIMESTEPS_NML
  TIMESTEPS%DTMAX = 900.  ! maximum global time step (s)
  TIMESTEPS%DTXY  = 300.  ! maximum CFL time step for x-y (s)
  TIMESTEPS%DTKTH = 450.  ! maximum CFL time step for k-th (s)
  TIMESTEPS%DTMIN =  30.  ! minimum source term time step (s)
/
&GRID_NML
  GRID%NAME        =  'G0816M2Sv3'
  GRID%NML         =  'namelist.nml'
  GRID%TYPE        =  'RECT'
  GRID%COORD       =  'SPHE'
  GRID%CLOS        =  'SMPL'
  GRID%ZLIM        =  -0.1
  GRID%DMIN        =  2.0
/
! NX,NY,SX,SY from coarsest SMC tier (smc_grid.inp)
&RECT_NML
  RECT%NX          =  2880
  RECT%NY          =  1200
  RECT%SX          =  0.125
  RECT%SY          =  0.125
  RECT%X0          = -179.96875
  RECT%Y0          =  -74.96875
/
! -------------------------------------------------------------------- !
! Define the spherical multiple-cell grid via SMC_NML namelist
&SMC_NML
  SMC%MCELS%FILENAME       =  'G0816Cell.dat'
  SMC%ISIDE%FILENAME       =  'G0816ISide.dat'
  SMC%JSIDE%FILENAME       =  'G0816JSide.dat'
  SMC%SUBTR%FILENAME       =  'G0816Obs.dat'
/
! -------------------------------------------------------------------- !
&OUTBND_COUNT_NML
  OUTBND_COUNT%N_LINE    = 4
/
&OUTBND_LINE_NML
  OUTBND_LINE(1)       = 163.2421875 -48.515625  0.029296875  0.000000000 628
  OUTBND_LINE(2)       = 181.6406250 -48.515625  0.000000000  0.019531250 904
  OUTBND_LINE(3)       = 181.6406250 -30.859375 -0.029296875  0.000000000 628
  OUTBND_LINE(4)       = 163.2421875 -30.859375  0.000000000 -0.019531250 904
/
EOF
```

namelist.nml

```
&PSMC DTIME = 20000.0, LvSMC=2, AVERG=F /
&FLX4 CDFAC = 1.00 /
&MISC CICE0=0.25, CICEN=0.75, FLAGTR=4, WCOR1=20.00, WCOR2=0.90 /
&SIN4 BETAMAX=1.75 /
&SIN6 SINA0 = 0.04  /
&SNL1 LAMBDA=0.237, NLPROP=2.13E+07 /
&SWL6 SWLB1 = 0.220E-03, CSTB1 =  T /
END OF NAMELISTS
```
